# A preliminary study on the application of electrical impedance tomography based on cerebral perfusion monitoring to intracranial pressure changes

**DOI:** 10.3389/fnins.2024.1390977

**Published:** 2024-05-28

**Authors:** Xiaoheng Yan, Yu Wang, Weichen Li, Mingxu Zhu, Weice Wang, Canhua Xu, Kun Li, Benyuan Liu, Xuetao Shi

**Affiliations:** ^1^Faculty of Electrical and Control Engineering, Liaoning Technical University, Huludao, China; ^2^Belt and Road Joint Laboratory on Measurement and Control Technology, Huazhong University of Science and Technology, Wuhan, China; ^3^College of Life Sciences, Northwest University, Xi’an, China; ^4^Department of Biomedical Engineering, Air Force Medical University, Xi’an, China

**Keywords:** electrical impedance tomography, cerebral perfusion, Valsalva maneuver, intracranial pressure, hemodynamic

## Abstract

**Background:**

In intracranial pathologic conditions of intracranial pressure (ICP) disturbance or hemodynamic instability, maintaining appropriate ICP may reduce the risk of ischemic brain injury. The change of ICP is often accompanied by the change of intracranial blood status. As a non-invasive functional imaging technique, the sensitivity of electrical impedance tomography (EIT) to cerebral hemodynamic changes has been preliminarily confirmed. However, no team has conducted a feasibility study on the dynamic detection of ICP by EIT technology from the perspective of non-invasive whole-brain blood perfusion monitoring. In this study, human brain EIT image sequence was obtained by *in vivo* measurement, from which a variety of indicators that can reflect the tidal changes of the whole brain impedance were extracted, in order to establish a new method for non-invasive monitoring of ICP changes from the level of cerebral blood perfusion monitoring.

**Methods:**

Valsalva maneuver (VM) was used to temporarily change the cerebral blood perfusion status of volunteers. The electrical impedance information of the brain during this process was continuously monitored by EIT device and real-time imaging was performed, and the hemodynamic indexes of bilateral middle cerebral arteries were monitored by transcranial Doppler (TCD). The changes in monitoring information obtained by the two techniques were compared and observed.

**Results:**

The EIT imaging results indicated that the image sequence showed obvious tidal changes with the heart beating. Perfusion indicators of vascular pulsation obtained from EIT images decreased significantly during the stabilization phase of the intervention (*PAC:* 242.94 ± 100.83, *p* < 0.01); perfusion index which reflects vascular resistance increased significantly in the stable stage of intervention (*PDT:* 79.72 ± 18.23, *p* < 0.001). After the intervention, the parameters gradually returned to the baseline level before compression. The changes of EIT indexes in the whole process are consistent with the changes of middle cerebral artery velocity related indexes shown in TCD results.

**Conclusion:**

The EIT image combined with the blood perfusion index proposed in this paper can reflect the decrease of cerebral blood flow under the condition of increased ICP in real time and intuitively. With the advantages of high time resolution and high sensitivity, EIT provides a new idea for non-invasive bedside measurement of ICP.

## 1 Introduction

Intracranial pressure (ICP) monitoring is an indispensable part of clinical care for many life-threatening brain injuries such as intracerebral hemorrhage, subarachnoid hemorrhage and malignant stroke. Clinical studies have shown that acute intracranial hypertension can even be life-threatening. Timely and accurate reflection of ICP can effectively prevent cerebral hernia and other lesions. On the other hand, cerebral perfusion pressure calculated by ICP can also determine the self-regulation state of cerebral vessels (Hawryluk et al., 2022). Therefore, ICP monitoring has very important clinical application value in the diagnosis and treatment of craniocerebral diseases.

At present, ICP monitoring is usually divided into two types: invasive measurement and non-invasive evaluation. Ventricular catheter is the current gold standard for ICP measurement, and it allows therapeutic drainage of cerebrospinal fluid (Valle et al., 2022). However, changes in the position of the ventricular catheter may reduce the accuracy of ICP measurement. In addition, placement of ventricular catheters can lead to severe bleeding and complications of infection (ventriculitis and meningitis) (Megjhani et al., 2022). The second commonly used device is the intraparenchymal transducer. It is divided into two categories: varistor based solid-state devices or fiber optic design devices (Aquilina et al., 2011). The overall safety of the intraparenchymal transducers is well, and there are few clinical complications related to infection and hematoma. However, on the other hand, this sensor cannot be recalibrated after placement, and although the insertion stage is very accurate, the measured value will drift to a certain extent over time (Al-Tamimi et al., 2009). Noninvasive ICP estimation methods such as optic nerve sheath diameter measurement (Tian et al., 2023), transcranial Doppler (TCD) measurement of blood flow velocity and automated pupillometry are increasingly used in the field of noninvasive ICP measurement (Giede-Jeppe et al., 2021; Martínez-Palacios et al., 2023). However, due to the high specificity between researchers and study populations, such methods require standardized training for operators. This makes non-invasive ICP measurement not enough to replace invasive ICP detection methods, either in terms of measurement accuracy or professional operation. Accurate, intuitive and dynamic measurement of ICP has become an urgent need to further improve the level of clinical brain disease treatment.

Electrical impedance tomography (EIT) is a new non-invasive and harmless imaging technology. It applies a safe current to the excitation electrode on the target surface, measures the boundary voltage difference between the remaining electrode pairs, calculates the inverse problem, and then reconstructs the time (or frequency) sequence image to directly reflect the impedance changes in the target region (Adler and Boyle, 2017). The change of ICP is often accompanied by the change of intracranial blood condition, and the resistivity of human blood is obviously different from that of other tissues. EIT technology is very sensitive to resistivity changes, and can be continuously monitored by imaging for a long time, which meets the requirements of blood flow change detection (Wang et al., 2022). Manwaring et al. (2013) proposed a domestic pig head injury model and designed a novel ICP/EIT electrode combination sensor. The combination of the conductivity difference image and ICP data has been used for the first time to monitor intracranial injury *in vivo* in real time. In the previous clinical study, Yang et al. in our research group showed that EIT can monitor the changes of brain water content related to brain edema in real time and non-invasively, and can detect brain edema early. During dehydration treatment, changes in EIT and ICP showed a close negative correlation (Yang et al., 2019). These studies confirm that base impedance changes can be used to reflect ICP status, and also accumulate the feasibility basis for EIT technology to be applied to non-invasive ICP monitoring. However, because the EIT base impedance is susceptible to dynamic interference, small movements of the subject may cause large fluctuations in the base impedance, which will affect the accuracy of indirect ICP measurement. Shi et al. (2018) and Li et al. (2019) of the group developed the EIT system, improved the accuracy and speed of data collection, and adopted the parallel EIT system to realize the imaging of the blood exchange process between the heart and lung. The real-time monitoring signal of dynamic blood perfusion can effectively avoid the interference caused by the subject’s movement and external operation, which provides a new idea for dynamic EIT technology to reflect the change of ICP in real time based on the cerebral blood perfusion state.

Based on the previous research on brain EIT measurement technology and imaging algorithm of our team, in this study, we focused on the monitoring of cerebral blood perfusion status during Valsalva maneuver (VM) intervention, and TCD as a control to study the feasibility and evaluation methods of EIT monitoring of changes in cerebral dynamic blood perfusion status and ICP monitoring. In order to explore the rule of ICP status and acute changes of intracranial perfusion through perfusion monitoring index extracted by impedance blood flow signal and reconstructed image non-invasively.

## 2 Materials and methods

### 2.1 Study protocol

This study was approved by the Ethics Committee of the Air Force Medical University [FMMU-E-III-001(1–7)]. All 12 recruited volunteers (9 males and 3 females, age 27.50 ± 3.34) signed written informed consent. Inclusion criteria were healthy volunteers with no history of brain disease and individuals with bilateral middle cerebral artery (MCA) asymmetrical flow velocity, arrhythmias, pulmonary ventilation disorders, and carotid artery stenosis were excluded. The experiment was completed in a room with good ventilation and convection. Subjects were prohibited from smoking and drinking alcohol or caffeine within 12 h prior to the experiment.

VM can induce cerebrovascular resistance (CVR) in the order of seconds, through which the brain automatically adjusts the vascular characteristics to comply with the acute changes of ICP or blood perfusion, so as to maintain the healthy neurovascular responsiveness of the body (Gomez et al., 2024). Therefore, measuring CVR has been used as a non-invasive tool to assess ICP indirectly. During VM intervention, the thoracic pressure will be increased and the brain will be in a state of intracranial hypertension, which will further affect the intracranial blood circulation (Pennacchietti et al., 2023). Before the experiment, each subject was familiar with VM under the guidance of the physician. Select a pressure gauge with a range of 10 kPa to measure the standard pressure required for VM quantitative measurement. After confirming that there was no air leakage, the physician instructed the subject to inhale deeply and exhale forcefully to form a pressure of about 40 mmHg (5.33 kPa) to ensure the accurate implementation of the intervention measures in the experiment.

Before the experiment, subjects washed their hair and kept their scalp dry to complete the attachment of the EIT electrode. Two TCD probes (KJ-2V7M, Kejin Industrial, Nanjing, China) were fixed at bilateral temporal fenestral locations, respectively, to monitor bilateral MCA hemodynamic parameters. The subjects remained seated throughout the experiment. After the experiment began, the subjects first rested for about 10 min, so that the electrical impedance of the brain and the TCD monitoring results remained stable. Subsequently, the subjects spontaneously completed VM intervention lasting about 20 s. After that, the subjects remained in the resting state for about 5 min until the end of the experiment. The whole process is measured continuously. During the experiment, if the subject feels unwell, the intervention will be stopped immediately and the experiment terminated.

### 2.2 Data collection

The acquisition of EIT signals adopts a new joint development EIT system (EC-100 PRO, UTRON Technology Co., Ltd., Hangzhou, China), which is based on the high-speed and high-precision system developed by our team in 2019 (Li et al., 2019). When the system is in the frequency range of 10–250 kHz, the signal to noise ratio can reach 90 dB at the frame rate of 40 fps.

**Figure 1:**
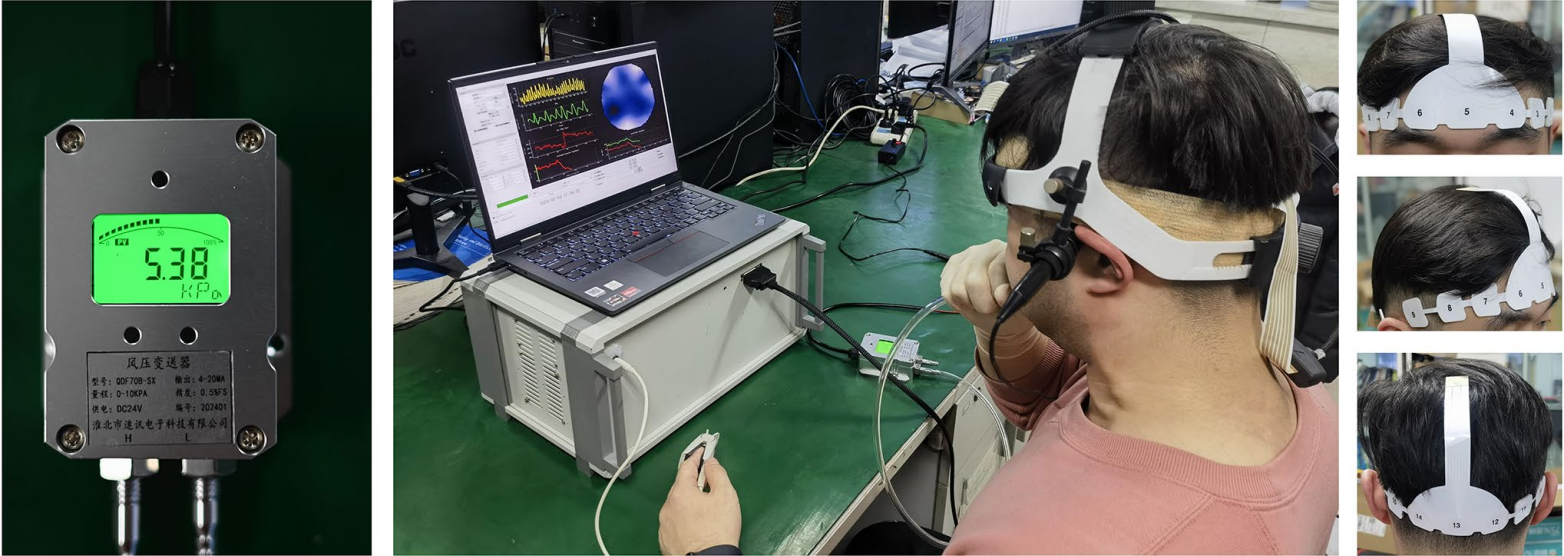
Experimental environment of EIT cerebral blood perfusion monitoring under VM intervention. The leftmost image showed the expiratory pressure (about 40 mmHg, 5.33 kPa) required to maintain the VM. The objects in the middle figure showed a subject’s experimental environment (including EIT and TCD equipment and real-time expiratory pressure display). The rightmost image showed the wear of EIT brain electrode band. (EIT, electrical impedance tomography; VM, Valsalva maneuver).

The system uses a disposable EIT electrode tape (EH-PET-16-CS, UTRON Technology Co., Ltd., Hangzhou, China) with 16 electrodes attached to the subject’s head. At the same time, the medical elastic bandage was tightly wrapped around the electrode to temporarily block the influence of scalp blood flow while assisting in fixing the electrode. The EIT data acquisition system we developed also integrates a photoplethysmography (PPG) signal acquisition channel. The two signals can be collected simultaneously, not separately by the two devices, which avoids the acquisition delay problem. Data were collected from the resting period of the subjects, and PPG signal of the subjects’ fingers were recorded synchronously during the collection, which was used as a reference for heart activity during the experiment. The experiment adopted a opposite 50 kHz current excitation mode, with a current effective value of 4 mA. The overall experimental process is shown in Figure 1.

### 2.3 Data analysis

All EIT data will be reconstructed using damped least squares algorithm (Xu et al., 2011). The reconstructed model is a finite element model based on one human brain CT image (including scalp, skull and brain parenchyma). The 12 volunteers used shared the same model for brain EIT imaging reconstruction. After image reconstruction, according to each EIT image obtained, the variation rate of average reconstruction impedance (*VR_ARI_*) of the whole brain was calculated using Eq. 1:


(1) $$VR_{ARI}=\left(\sum_{i=1}^{N}\Delta \rho_{i}\right)/N$$

In Eq. 1, ∆*ρ_i_* represents the variation rate related to the resistivity of the *i*-th pixel in each EIT image and *N* represents the total number of pixels; the unit of *VR_ARI_* is arbitrary unit (a.u.).

We defined the peak moment of the waveform in each cycle as the starting point of the cerebral perfusion cycle, and the value at this time was denoted as *VR_P_*. *VR_ARI_* in each cycle was analyzed, and relevant indexes were extracted to evaluate the changes of cerebral blood flow during the experiment.

Pulse Delay Time (*PDT*) was calculated by Eq. 2:


(2) $$PDT=M_{PPG}-M_{VR}$$

*M_PPG_* is the moment when the changing rate of the ascending branch of PPG signal is the largest in each perfusion cycle, and *M_VR_* is the moment when the changing rate of the descending branch of *VR_ARI_* is the largest in the same perfusion cycle. The unit of *PDT* is millisecond (ms).

Perfusion Area per Cycle (*PAC*) was calculated by Eq. 3:


(3) $$PAC=-\frac{1}{N}\sum_{i=1}^{N}\left(VR_{ARI}\left(i\right)-VR_{P}\right)$$

*N* is the total number of EIT image frames obtained during the current perfusion cycle, and *VR_ARI_*(i) is the average reconstruction impedance change rate of EIT image in the *i*-th frame; the unit of *PAC* is arbitrary unit (a.u.).

Statistical analyses were executed with SPSS version 27.0 (SPSS Inc., Chicago, IL, United States). To assess the normality of each variable’s distribution, the Shapiro–Wilk test was employed. The data have been represented either as the mean value (± standard deviation) when the criteria for Gaussian distribution were fulfilled or as the median (with interquartile range) in cases where normality was not met. To compare the perfusion indices derived from the baseline and intervention phases, we utilized either a paired *t*-test or the Wilcoxon signed-rank test, contingent upon the fulfillment of normality assumptions. The threshold for statistical significance was established at *p* < 0.05.

## 3 Results

### 3.1 EIT image reconstruction

**Figure 2:**
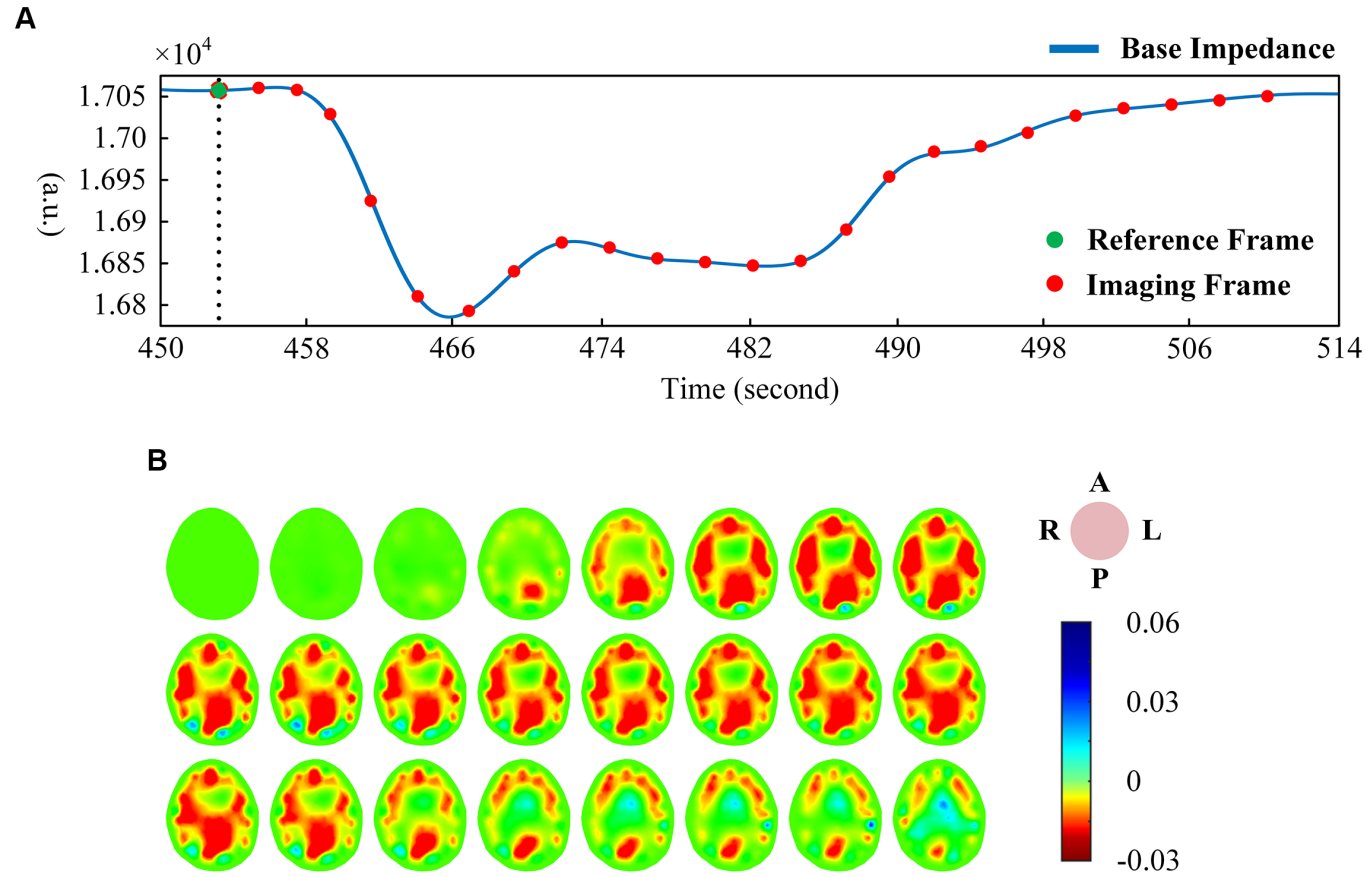
**(A)** One-dimensional curve change of base impedance during VM intervention; **(B)** Reconstructed image of EIT base impedance (interval 2 s). (EIT, electrical impedance tomography; VM, Valsalva maneuver).

The EIT signal was first smoothed using a low-pass filter (about *f* = 3 Hz), and then the smoothed signal was low-pass filtered (about *f* = 0.01 Hz) to obtain the base impedance signal reflecting the trend change. Figure 2A shows the change of base impedance of a typical subject, from which it can be seen that the base impedance drops sharply at the beginning of VM. It then recovered to a plateau. At the end of the intervention, the base impedance recovery changed from fast to slow, and finally returned to the baseline state. The voltage data before VM intervention was used as reference frame, and one frame of voltage data was selected as foreground frame every 2 s for image reconstruction of base impedance. As can be seen in Figure 2B (the same subject), under the condition of sharp decline in base impedance, intracranial blood increased, corresponding to large expansion of the red region of the whole brain (the optimal filling state was reached at the lowest point). Subsequently, the intracranial blood gradually decreased and returned to the baseline state according to the curve change trend.

**Figure 3:**
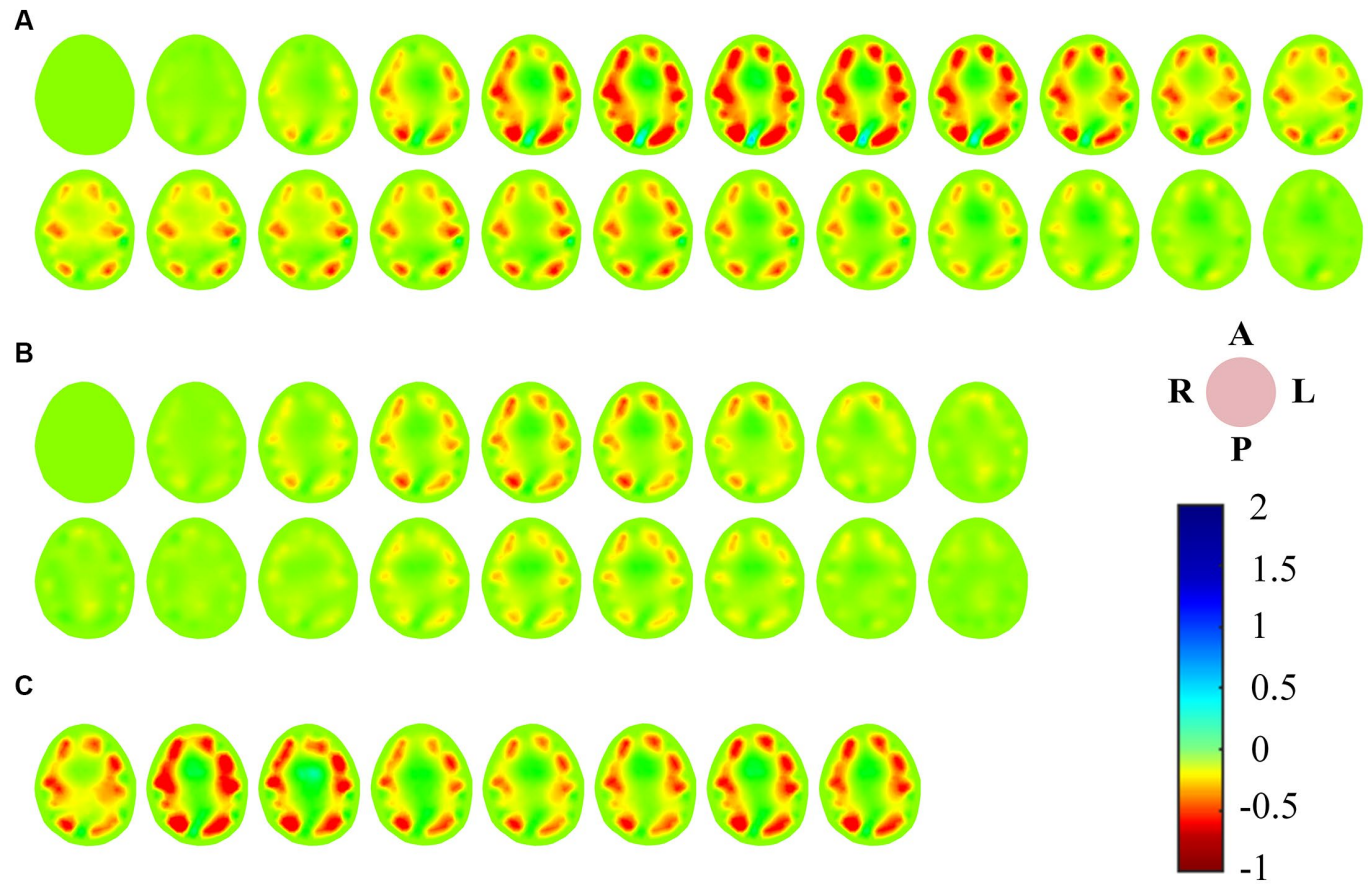
**(A)** EIT images of a single perfusion cycle during BP; **(B)** EIT images of a single perfusion cycle during IP; **(C)** A single perfusion cycle was selected every 2 s to obtain EIT images at the time of maximum cerebral blood flow perfusion. (EIT, electrical impedance tomography).

There was a good correlation between the tidal changes of cerebral blood perfusion signal filtered from the original impedance signal and the cardiac activity shown by PPG. In the stable phase of baseline period (BP) and intervention period (IP), the wave crest of perfusion signal was selected as the reference frame, and the data of the cycle after the reference frame was used as the reconstructed frame for image reconstruction, so that each volunteer could obtain repeatable continuous imaging results. Figure 3A shows the EIT image of a single perfusion cycle in the BP stage. At the beginning of perfusion, the resistivity of the central region of the brain decreased slightly (the image showed a light yellow change), and the degree and area of the resistivity change gradually expanded, indicating that blood began to flow into the brain. From 50 to 175 ms, the resistivity of the peripheral region decreased rapidly, resulting in a rapid dark red change in the corresponding area of the image, corresponding to the rapid perfusion of blood into the skull. The optimal filling state of cerebral blood perfusion was achieved at 175 ms. During the subsequent 200–350 ms period, the electrical resistivity of the peripheral brain region began to rise rapidly (the red color quickly lightened). During the 375–450 ms period, the electrical resistivity of the peripheral region decreased slightly (the color depth of the red region increased slightly), suggesting a slight increase in cerebral blood flow in the corresponding region. After that, the resistivity of each area gradually recovered to the initial level, indicating the intracranial blood flow back to the heart and the end of a perfusion cycle. The contents shown in Figures 2, 3 were from the same subject.

For the single-cycle cerebral blood perfusion images in the IP stage (Figure 3B), the duration was shorter than that in the BP, and the perfusion intensity was also somewhat weakened. From 25 to 125 ms after the reference frame, the electrical resistivity of the peripheral brain region gradually decreased, and the corresponding area of the image turned red in small areas. From 150 to 275 ms, the whole brain resistivity quickly returned to a state close to the initial level. During the subsequent 75 ms period, the resistivity of the peripheral region decreased slightly. Cerebral blood perfusion filled briefly and then quickly returned to the initial level.

In addition, we selected an perfusion cycle every 2 s during the VM intervention stage to obtain images at the moment of maximum cerebral blood perfusion, as shown in Figure 3C. Excluding the disturbance caused by movement, the peak cerebral blood flow increased first, then decreased, and finally returned to a stable state during the effective intervention period of 20 s.

### 3.2 Index analysis of EIT images

**Figure 4:**
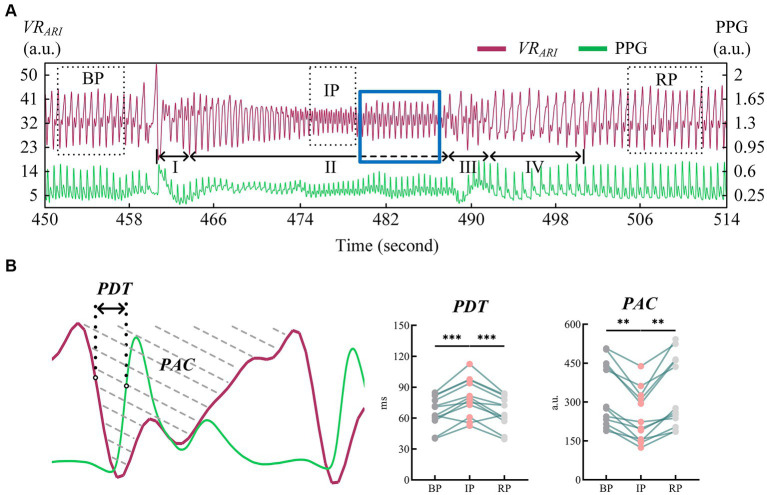
**(A)** Cerebral blood perfusion curves extracted from EIT reconstructed images were compared with PPG signals; **(B)** Results of statistical analysis of EIT cerebral blood perfusion monitoring indexes. (EIT, electrical impedance tomography; PPG, photoplethysmography). ** means *p* < 0.01. *** means *p* < 0.001.

We refer to the period after VM intervention until the end of the experiment as RP. Ten consecutive perfusion cycles were taken from each of the three periods of BP, IP and RP (Figure 4A), and indexes were extracted from the *VR_ARI_* results of these periods to calculate the mean value of cerebral blood perfusion indexes. The data of all subjects were statistically analyzed, as shown in Table 1. It can be seen that for *PDT* index, the value of IP was greater than that of BP and RP, and there was a statistically significant difference. In addition, the *PAC* of IP segment was significantly smaller than that of BP and RP. The statistical analysis between BP and RP showed that there was no significant difference between the two indexes. It was suggested that the perfusion status of the whole brain returned to normal after VM intervention (Figure 4B).

**Table 1:** Effects of VM on global cerebral perfusion monitoring using EIT.

Index	Period	Variable	Trend-*p* value		
			BP	IP	RP
*PDT* (ms)	BP	66.07 ± 15.02	\	< 0.001	0.987
	IP	79.72 ± 18.23		\	< 0.001
	RP	66.07 ± 15.09			\
*PAC* (a.u.)	BP	278.55 [219.16–446.31]	\	< 0.01	0.239
	IP	242.94 ± 100.83		\	< 0.01
	RP	268.52 [211.80–463.21]			\

### 3.3 Index analysis of TCD measurement

TCD measured the mean blood flow velocity (*V_m_*), pulsation index (*PI*), resistance index (*RI*) and the ratio of systolic to diastolic blood flow velocity (*S/D*) in the bilateral MCAs during the experimental period. There was no significant difference between the left and right indexes of BP, IP and RP. Therefore, we used the mean value of the left and right MCA indicators as a statistic for analysis. Table 2 shows the changes of MCA blood flow velocity recorded by the TCD device in 12 subjects during the experiment.

**Table 2:** Effects of VM on global cerebral perfusion using TCD.

Index	Period	Variable	Trend-*p* value		
			BP	IP	RP
*V_m_* (cm/s)	BP	53.88 ± 10.35	\	< 0.001	0.897
	IP	33.83 ± 5.59		\	< 0.001
	RP	53.79 ± 10.06			\
*PI* (a.u.)	BP	0.91 ± 0.17	\	< 0.001	0.410
	IP	1.53 ± 0.38		\	< 0.01
	RP	0.94 [0.83–1.00]			\
*RI* (a.u.)	BP	0.66 [0.63–0.70]	\	< 0.01	0.964
	IP	0.78 ± 0.10		\	< 0.01
	RP	0.66 [0.64–0.68]			\
*S/D* (a.u.)	BP	2.20 ± 0.27	\	< 0.001	0.610
	IP	3.37 ± 0.84		\	< 0.01
	RP	2.21 [2.08–2.35]			\

**Figure 5:**
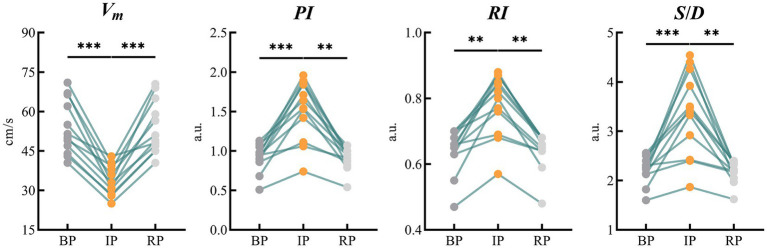
Results of statistical analysis of blood flow parameters of bilateral MCAs monitored by TCD. (MCA, middle cerebral artery; TCD, transcranial Doppler). ** means *p* < 0.01. *** means *p* < 0.001.

All indicators were consistent in both BP and RP periods, and there was no significant statistical difference between the two periods. In IP period, *V_m_* decreased significantly compared with BP and RP period. The values of *PI* and other indexes were lower than those of BP and RP. These differences were statistically significant (Figure 5).

## 4 Discussion

Real-time dynamic brain ICP non-invasive monitoring has important application prospects in the basic research of brain science, the early diagnosis of brain diseases, and the warning of major changes in brain injuries. The modern ICP measurement technology represented by invasive and non-invasive has some shortcomings, and it is difficult to fully meet the clinical needs. Compared with these technologies, EIT has outstanding advantages in non-invasive technology, easy operation, imaging sensitivity and high speed characteristics, so as to better make up for the shortcomings of existing technologies. Changes in intracranial pressure can lead to changes in cerebral blood flow status. Theoretically, EIT technology can detect cerebral blood perfusion status and reflect ICP status based on the biophysical basis that blood resistivity is much lower than brain tissue. However, on the one hand, the change rate of electrical impedance caused by changes in blood flow is very weak; on the other hand, the high resistivity characteristics of the skull greatly hinder the collection of intracranial impedance information, which makes it very difficult to detect intracranial cerebral blood flow changes from the scalp surface (Ke et al., 2022).

Based on the team’s research foundation in the field of brain EIT and the latest research progress in high-performance data collection technology, to explore the feasibility of dynamic monitoring on cerebral blood perfusion reflecting ICP by EIT, this study for the first time aimed at non-invasive ICP monitoring, observed changes in brain impedance during resting and VM intervention through EIT data collection and image reconstruction, and studied the correlation between EIT imaging results and brain blood supply process. For the goal on quantitative evaluation of EIT monitoring results, *PAC* was extracted according to the influence of cerebral blood perfusion volume on blood flow velocity, and *PDT* was extracted according to the influence of cerebrovascular pulsation and resistance to evaluate the delay of blood supply.

In order to observe the EIT image of cerebral blood perfusion status during the change of ICP and the performance of monitoring indicators extracted from the image, we adopted VM mode, which is commonly used in clinical regulation of autonomic nervous function, and gradually expanded its application in the diagnosis and treatment of nervous system diseases. The changes in blood pressure induced by VM are operable, safe and accurate, making it widely used in healthy people and clinical patients (Lee et al., 2015). VM affects blood circulation by increasing intrapleural pressure, resulting in increased ICP and decreased cerebral perfusion pressure. Based on this feature, we took it as the intervention action in this experiment, and studied the corresponding cerebral blood perfusion changes according to this ICP increase model, thus verifying the feasibility of EIT monitoring cerebral blood perfusion for real-time dynamic association with ICP.

Classic VM are typically divided into four phases (Roldan et al., 2023). In stage I, the abdominal force is exerted when the breath is held after deep inspiration, and the closed airway is forcefully exhaled. The sudden increase of intrapleural pressure compresses the aorta, resulting in a brief increase of arterial blood pressure (ABP) and blood transfer to the peripheral artery. Stage II is expiratory maintenance. The increase of intrathoracic pressure prevents venous return to the heart, peripheral venous pressure gradually increases, cardiac output decreases, ABP decreases, and heart rate increases. Stage III is expiratory release, the intrathoracic pressure decreases, the return blood volume increases, the peripheral arterial pressure decreases, and the heart rate increases. Stage IV venous return recovery leads to diastolic heart filling and gradual recovery of ABP and heart rate. From the measured EIT cerebral perfusion results, it can be seen that in stage I, the amplitude of cerebral blood flow impedance signal increased correspondingly due to the sudden increase of ABP. When ABP gradually decreased and ICP continuously increased in stage II, cerebral blood flow decreased, and the shape of blood perfusion wave changed greatly, and the dicrotic wave disappeared. This may be due to the blockage of vena cava return caused by increased intrapleural pressure, which in turn leads to impaired atrial filling and changes in cardiac blood supply mechanism (Knauth et al., 2017). When the ICP caused by VM rose to the highest level, the cerebral blood perfusion status as shown in IP stabilized and the shape changed again. The reason for this phenomenon may be stimulated by decreased ABP, which leads to increased heart rate and sympathetic nerve excitation, and new changes in the heart blood supply mechanism (Haykowsky and Eves, 2003). We extended the duration of stage II in the experiment, as shown by the dotted line (blue rectangular box). It may be because the self-regulation of cerebrovascular triggers the brain compensation mechanism, and the waveform of this stage gradually returns to normal, similar in shape to that of BP stage, but still smaller in amplitude than that of BP stage. At the beginning of stage III, the abdominal muscles begin to relax and the intrapleural pressure drops rapidly, leading to another brief drop in ABP. At this stage, the cerebral impedance blood flow waveform involved body motion interference, and the quality was poor and could not be described qualitatively. In stage IV, the decrease of intrapleural pressure impeded the return of vena cava and restored the damaged atrial filling, stimulated vagus nerve, and slowed down the heart rate. In the corresponding stage, cerebral blood flow gradually returned to the normal state, and the position of dicrotic wave in the impedance blood flow waveform was gradually restored in each cycle. The EIT reconstructed images showed that the blood perfusion in the steady-state stage of VM intervention was significantly less than that in BP stage. The volume of cerebral perfusion per period was also different in different stages of VM, which was consistent with the physiological phenomenon of specific stage under VM intervention. In addition, the value of *PDT* in the IP period was significantly higher than that in the baseline period (79.72 ± 18.23, *p* < 0.001). The value of *PAC* was lower than the baseline period, and the difference was statistically significant (242.94 ± 100.83, *p* < 0.01). At the same time, the blood flow data of bilateral cerebral arteries of TCD jointly monitored showed that the cerebrovascular velocity decreased and the vascular resistance and pulsation index increased during VM, with statistical significance (*p* < 0.01), which was in good accordance with the rule of EIT cerebral perfusion monitoring.

In previous studies on EIT brain measurement combined with ICP, animal models were often used to carry out relevant experiments. As the brain structure of domestic pigs or rabbits is quite different from that of human beings, the research results may be different from the results of human experiments (Manwaring et al., 2013; Zhang et al., 2022). In addition, most studies use the operation method of contrast agent, relying on reagents to reach the brain through blood circulation to achieve the goal of contrast agent enhanced imaging (Yang et al., 2019; Zhang et al., 2023). However, arteriography perfusion has some disadvantages such as large trauma and inconvenient operation, so it cannot be used repeatedly in human experiments. The technical method of this study only needs to attach electrodes to the human scalp surface to carry out the study, without the use of contrast agents or other drugs, avoiding interference from other factors, and only monitoring intracranial blood perfusion.

The limitations of this study are as follows: (1) The research was still in the qualitative stage, and the subjects were all healthy young volunteers. Whether the relevant results are applicable to other populations needs to be further verified. (2) Based on the goal of complete non-invasive and real-time control, TCD technology in this study can only provide relevant information about the blood flow velocity of the target blood vessel, and cannot further determine the actual perfusion of the whole brain. In further research, a systematic comparative analysis with ventricular catheter ICP measurement method can be considered in the clinical environment to establish the quantitative relationship between EIT parameters and actual ICP measurements (Rubin and Berra, 2022); (3) The *PDT* extracted in this study to reflect the vascular resistance and propagation delay time is based on finger pulse wave as a reference, which may be affected by the delay of blood transmission at the arm. Electrocardiogram or heart sound signals may be considered to further improve the accuracy of indicator measurement. (4) The drop in blood pressure that occurs during the execution of the VM is predominantly because of decreased venous return, which results from elevated intrathoracic pressure. Consequently, this effect might not genuinely mirror the variations in blood pressure associated with vascular resistance encountered in real-world clinical situations. The parameters related to the time of arrival of the pulse still need to be refined.

## 5 Conclusion

This study used EIT to reflect the changes of cerebral blood perfusion under VM intervention, which provided a new idea for ICP monitoring. The results showed that EIT reconstructed images visually demonstrated the changes of intracranial blood perfusion status during the rise of ICP, and the perfusion monitoring indexes extracted from the reconstructed images could accurately describe the differences of cerebral blood perfusion status under different ICP environments. Therefore, EIT is expected to make up for the shortage of existing technologies and play an important role in many fields of clinical medicine and basic science, such as diagnosis of craniocerebral critical diseases, early warning and monitoring of brain injury, intraoperative brain protection, and neuroregulation assessment of major operations.

## Data availability statement

The raw data supporting the conclusions of this article will be made available by the authors, without undue reservation.

## Ethics statement

The studies involving humans were approved by the ethics review board of Air Force Medical University (Trial registration: ChiCTR-OOC-16007844; registered on 23 November 2022). The studies were conducted in accordance with the local legislation and institutional requirements. The participants provided their written informed consent to participate in this study. Written informed consent was obtained from the individual(s) for the publication of any potentially identifiable images or data included in this article.

## Author contributions

XY: Funding acquisition, Supervision, Validation, Writing – review & editing. YW: Data curation, Formal analysis, Investigation, Methodology, Visualization, Writing – original draft, Writing – review & editing. WL: Conceptualization, Data curation, Investigation, Project administration, Supervision, Writing – review & editing. MZ: Data curation, Formal analysis, Software, Writing – review & editing. WW: Data curation, Formal analysis, Supervision, Writing – review & editing. CX: Methodology, Project administration, Software, Visualization, Writing – review & editing. KL: Resources, Validation, Writing – review & editing. BL: Resources, Software, Supervision, Validation, Visualization, Writing – review & editing. XS: Conceptualization, Funding acquisition, Project administration, Software, Supervision, Validation, Writing – review & editing.
